# The Role of Amino Acid Substitution in HepT Toward Menaquinone Isoprenoid Chain Length Definition and Lysocin E Sensitivity in *Staphylococcus aureus*

**DOI:** 10.3389/fmicb.2020.02076

**Published:** 2020-08-26

**Authors:** Suresh Panthee, Atmika Paudel, Hiroshi Hamamoto, Anne-Catrin Uhlemann, Kazuhisa Sekimizu

**Affiliations:** ^1^Teikyo University Institute of Medical Mycology, Hachioji, Japan; ^2^Department of Medicine, Columbia University Medical Center, New York, NY, United States

**Keywords:** menaquinone, *Staphylococcus aureus*, MRSA, mechanisms of action, HPLC, genomics, antimicrobial agents, lysocin E

## Abstract

**Objectives:**

*Staphylococcus aureus* Smith strain is a historical strain widely used for research purposes in animal infection models for testing the therapeutic activity of antimicrobial agents. We found that it displayed higher sensitivity toward lysocin E, a menaquinone (MK) targeting antibiotic, compared to other *S. aureus* strains. Therefore, we further explored the mechanism of this hypersensitivity.

**Methods:**

MK production was analyzed by high-performance liquid chromatography and mass spectrometric analysis. *S. aureus* Smith genome sequence was completed using a hybrid assembly approach, and the MK biosynthetic genes were compared with other *S. aureus* strains. The *hepT* gene was cloned and introduced into *S. aureus* RN4220 strain using phage mediated recombination, and lysocin E sensitivity was analyzed by the measurement of colony-forming units.

**Results:**

We found that Smith strain produced MKs with the length of the side chain ranging between 8 and 10, as opposed to other *S. aureus* strains that produce MKs 7–9. We revealed that Smith strain possessed the classical pathway for MK biosynthesis like the other *S. aureu*s. HepT, a polyprenyl diphosphate synthase involved in chain elongation of isoprenoid, in Smith strain harbored a Q25P substitution. Introduction of *hepT* from Smith to RN4220 led to the production of MK-10 and an increased sensitivity toward lysocin E.

**Conclusion:**

We found that HepT was responsible for the definition of isoprenoid chain length of MKs and antibiotic sensitivity.

## Introduction

Menaquinone (MK), found in the cytoplasmic membrane, is an essential component of the electron transport chain in Gram-positive bacteria. Apart from respiration, it plays vital roles in oxidative phosphorylation and the formation of transmembrane potential. Given the importance of MK in cellular survival, MK and its biosynthesis has been extensively studied (Hiratsuka et al., 2008; Seto et al., 2008; Paudel et al., 2016). It has been shown that MK analogs inhibit the bacterial growth (Schlievert et al., 2013) and several enzymes involved in MK biosynthesis such-as isoprenoid precursor (Jomaa et al., 1999); naphthoquinone (Fang et al., 2010); and incorporation of the isoprenoid side chain to naphthoquinone moiety (Dhiman et al., 2009) can independently be targeted for antimicrobial agent discovery against Gram-positive and acid-fast microbes. Recently, we reported that lysocin E, a non-ribosomally synthesized peptide (Panthee et al., 2016, 2017b) produced by *Lysobacter* sp. RH2180-5, directly targets MK in the bacterial membrane exerting rapid and potent bactericidal activity (Hamamoto et al., 2015).

MK is a 2-methyl-1,4-naphthoquinone with an isoprenoid side chain attached at the 3-position. MK is generally referred to as MK-n, where n denotes the number of isoprenoid units between 4 and 13 attached to the naphthoquinone core. The units of isoprene in the MKs differ among different species and sometimes even within the same species (Kurosu and Eeshwaraiah, 2010). The difference in MK isoprenoid chain formed a basis of bacterial chemotaxonomic identification in pre genomic era (Collins and Jones, 1981).

*Staphylococcus aureus* is a human commensal and an opportunistic pathogen responsible for a large number of hospitalization and deaths. Global spread and rise of methicillin-resistant (Moellering, 2011; Mostofsky et al., 2011) and vancomycin-resistant *S. aureus* strains (Mcguinness et al., 2017; Panthee et al., 2017a, c) have added the burden to health-care systems. *S. aureus* uses MKs with the length of the side chain ranging between 7 and 9, where MK-8 is the most predominant (Collins and Jones, 1981). *S. aureus* strain Smith, isolated in 1930, is widely used in the laboratory for the development of mouse infection model as it displays a high degree of virulence against mouse model (Hunt and Moses, 1958). Previously, we found that it displayed a higher susceptibility toward menaquinone targeting antibiotic-lysocin E (Hamamoto et al., 2015). This led to speculation that MK biosynthetic machinery in *S. aureus* Smith might be different from other *S. aureus*. In this study, we report the complete genome sequence, MK analysis of *S. aureus* Smith and the factor responsible for its hypersensitivity toward lysocin E. To the best of our knowledge, this is the first report of the identification of *S. aureus* strain producing MK-10, and the involvement of a single amino acid substitution in HepT for MK-10 production and sensitivity toward antibiotic.

## Materials and Methods

### Microorganisms and Culture Conditions

The bacterial strains and plasmids used in this study are summarized in Table 1. *S. aureus* strains were routinely grown on tryptic soy broth, and *Escherichia coli* was grown on Luria-Bertani medium. Antibiotics were supplemented to the medium as required.

**TABLE 1:** Bacterial strains and plasmids used in this study.

Strain/Plasmid	Details/Source
*Staphylococcus aureus*	
Smith	Isolated in 1930 (Hunt and Moses, 1958), ATCC13709
RN4220	Restriction deficient strain, laboratory stock
Newman	Isolated in 1952 (Duthie and Lorenz, 1952), laboratory stock
JE2	USA300 strain obtained from BEI Resources
71101	Clinical isolate (Uhlemann et al., 2017)
NCTC5663	Public Health England
*Escherichia coli* HST08	Competent cells for routine cloning from Takara

pND50-p*fbaA*	pND50 with *fbaA* promoter inserted in *Eco*RI/*Bam*HI site (Paudel et al., 2020)
pND50-p*fbaA-hepT*_Smith_	*hepT*_Smith_ in pND50-p*fbaA*
pND50-p*fbaA-hepT*_RN4220_	*hepT*_RN4220_ in pND50-p*fbaA*

### Whole-Genome Sequencing, Assembly, and Comparative Genomic Analysis

The complete genome of *S. aureus* Smith was sequenced using hybrid genome assembly as explained previously (Panthee et al., 2018, 2019; Paudel et al., 2019) using 1 μg and 100 ng of genomic DNA for Oxford Nanopore MinION and Thermo Fisher Scientific Ion PGM, respectively. The assembled genome was annotated using the NCBI Prokaryotic Genome Annotation Pipeline. The draft genome of *S. aureus* 71101 was obtained by Illumina sequencing (Uhlemann et al., 2017). The complete genome sequences of 324 *S. aureus* strains were obtained from NCBI GenBank, and amino acid sequences of MK biosynthetic genes were obtained using BLAST search.

### *hepT* Cloning and Heterologous Expression

The *hepT* gene from *S. aureus* was amplified using the primer sets BamF vs. SalR1 and BamF vs. SalR2 for Smith and RN4220 strains, respectively (Table 2). The *Bam*HI *Sal*I digested PCR product was then ligated to pND50-p*fbaA* vector (Paudel et al., 2020) digested with the same enzymes to construct pND50-p*fbaA-hepT*_Smith_ and pND50-p*fbaA-hepT*_RN4220_, respectively. The ligated plasmid was then transformed to *Escherichia coli* HST08 (Takara Bio) and selected on chloramphenicol plates. The strains with correct sequences were selected for transformation into electrocompetent *S. aureus* RN4220. Insertion in the RN4220 strain was then confirmed by PCR.

**TABLE 2:** Primers used to amplify *hepT* gene.

Primer	Sequence 5′–3′ (underline indicate the restriction site)
BamF	CGCGGATCCATGAACAATGAAATTAAGAA
SalR1	ACGCGTCGACAATACTATGTGTTTCTTGAC
SalR2	ACGCGTCGACCTACGTGTTTCTTGAACCCA

### Menaquinone Extraction and HPLC Analysis

*S. aureus* strains were cultured overnight in 5 mL TSB supplemented with antibiotics as required in a shaking incubator maintained at 37°C. The full growth was then diluted 100-fold in the 5 mL TSB medium without antibiotics and incubated in the same shaker for 16 h. A 300 μL of the culture broth was extracted twice with 1.5 mL of hexane 5: ethanol 2. The supernatant was pooled, dried *in vacuo*, dissolved in 200 μL ethanol and 80 μL of it was analyzed using a Waters Alliance high-performance liquid chromatography (HPLC) system equipped with a Senshu Pak PEGASIL ODS SP100 column (4.6φ × 250 mm) maintained at 40°C. After the application of the sample to the column equilibrated with 1 mL min^–1^ of 20% diisopropyl ether in methanol, the column was eluted with the same solvent. Detection was made using a fluorescent detector using wavelengths 320 and 430 nm for excitation and emission, respectively, after post-column reduction using a platinum column. The extraction efficiency of this protocol was confirmed by the analysis of MKs from *S. aureus* USA300 JE2 using ubiquinone – 10 (UQ-10) as an internal standard followed by the analysis of extracted UQ-10 using a PDA detector at 275 nm (Supplementary Figure S1).

### High Resolution Mass Spectrometric Analysis

High resolution mass spectrometric analysis was performed on a UPLC/MS system using a Waters Acquity UPLC consisting of 2.1 × 50 mm Acquity UPLC^®^ BEH C18 1.7 μm column. After the injection of the sample to the column equilibrated with 0.3 mL min^–1^ of 100% methanol, the eluate was continuously applied to a Waters Xevo G2-XS QTof mass spectrometer. The data at the mass range of 100–1700 Da were collected in ESI positive mode using a source capillary voltage of 2.00 kV. The data were obtained using MassLynx 4.1 (Waters Milford, MA, United States) and analyzed by UNIFI Scientific Information System (Waters).

### Lysocin E Susceptibility

Viability of *S. aureus* upon treatment with lysocin E was determined as described previously (Paudel et al., 2012, 2013, 2017) following NCCLS protocol (National Committee for Clinical Laboratory Standards, 1999). Briefly, the overnight full growth of *Staphylococci* was diluted 100-fold with 5 mL TSB and incubated at 37°C with shaking. After the OD_600_ reached 0.1, 1 mL aliquot was collected and treated with 1 mg/L of lysocin E, and incubation was continued for 30 min. The number of the surviving bacteria was counted by spreading on Mueller Hinton agar plates. Untreated samples at time zero were considered as 100% and used to calculate percentage survival.

## Results and Discussion

### Higher Sensitivity of *S. aureus* Smith Toward Lysocin E

**FIGURE 1:**
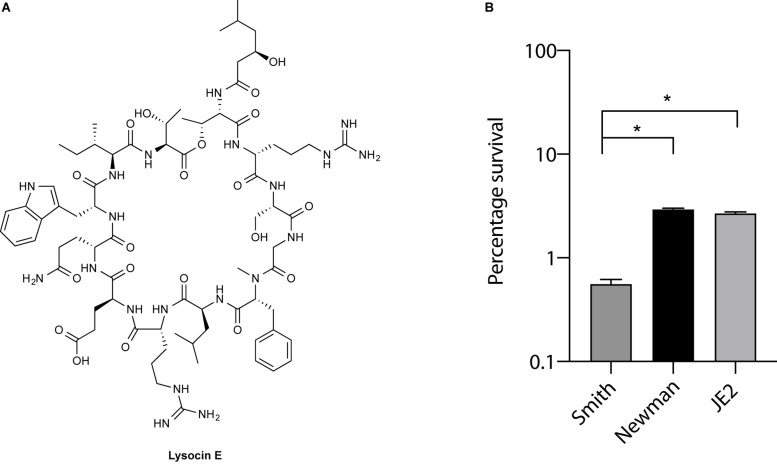
Lysocin E and its antimicrobial activity. **(A)** Chemical structure of lysocin E. **(B)** Bactericidal activity of lysocin E. *S. aureus* strains were treated with 1 mg/L lysocin E for 30 min, and bacterial viability was determined. Triplicate data are represented as mean ± SEM and statistical analysis was performed by one-way ANOVA using Dunnett’s multiple comparison test in GraphPad Prism. The asterisk indicates a *p*-value of < 0.0001.

**FIGURE 2:**
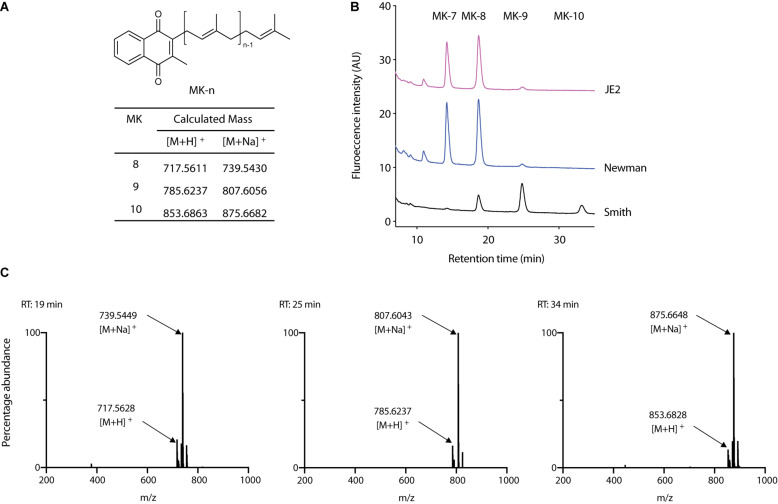
Analysis of MKs from *S. aureus*. **(A)** Chemical structure of MK-n and calculated exact mass of MK-8, 9, and 10 in positive ion analysis. **(B)** Analysis of MK extract from *S. aureus* Smith, Newman, and JE2. **(C)** High-resolution mass spectrometric analysis of peaks that appeared in Smith at 19, 25, and 34 min.

Lysocin E (Figure 1A) is a recently discovered antibiotic effective against Gram-positive bacteria that utilize MK for respiration (Hamamoto et al., 2015; Itoh et al., 2019). Lysocin E has a potent and rapid bactericidal activity. During our routine experiments, we found that *S. aureus* Smith strain was consistently more sensitive toward lysocin E with a 2-fold lower MIC value compared with other *S. aureus* strains. Since a 2-fold difference in MIC is usually regarded within the error range (Clinical and Laboratory Standards Institute, 2012), we performed much sensitive and quantitative assay by determining bactericidal activity of lysocin E against various *S. aureus* strains. We found a significantly higher bactericidal activity of lysocin E against Smith compared to Newman and JE2 strains (Figure 1B), suggesting its hypersensitive nature. As lysocin E targets MK (Hamamoto et al., 2015), and *S. aureus* has MK as the sole quinone known to be utilized for respiration (Bentley and Meganathan, 1982), we speculated that the MKs in Smith strain could be different from other *S. aureus* strains. However, there is no study about the type, content, and biosynthesis of MKs in *S. aureus* Smith. Therefore, we extracted MKs form the overnight cultures of the *S. aureus* Smith, Newman, and JE2 strains and analyzed by HPLC. Consistent with the previous report (Wakeman et al., 2012), Newman strain mainly produced MK-7 and MK-8, MK-8 being the most abundant, and trace amounts of MK-9. While MK production in JE2 was similar to that of Newman strain, Smith strain mainly produced MK-8 and MK-9, with MK-9 being the most abundant, and there appeared an undefined peak at the retention time of 34 min (Figures 2A,B). We then extracted MKs from a 50-mL volume of culture and separately collected each peak and analyzed by high-resolution mass spectrometry. We found that the peaks were 739.5449, 807.6043, and 875.6648 corresponding with [M + Na]^+^ of MK-8, MK-9, and MK-10, respectively (Figure 2C). The undefined peak was thus identified as MK-10. Therefore, as opposed to the major quinone MK-8 in *S. aureus* (Collins and Jones, 1981), Smith strain produced MK-9 predominantly. In addition, Smith strain produced MK-10, an MK that has not been reported in *S. aureus*. These results suggested that longer chain MKs in Smith strain might be responsible for its hypersensitivity toward lysocin E. Previously we found that *S. aureus* strains with mutation and/or deletions in the genes involved in MK biosynthesis were resistant to lysocin E (Hamamoto et al., 2015) suggesting that analysis of MK biosynthetic genes in Smith would give an insight upon its hypersensitivity.

### Analysis of MK Biosynthetic Pathway in *S. aureus* Smith

**FIGURE 3:**
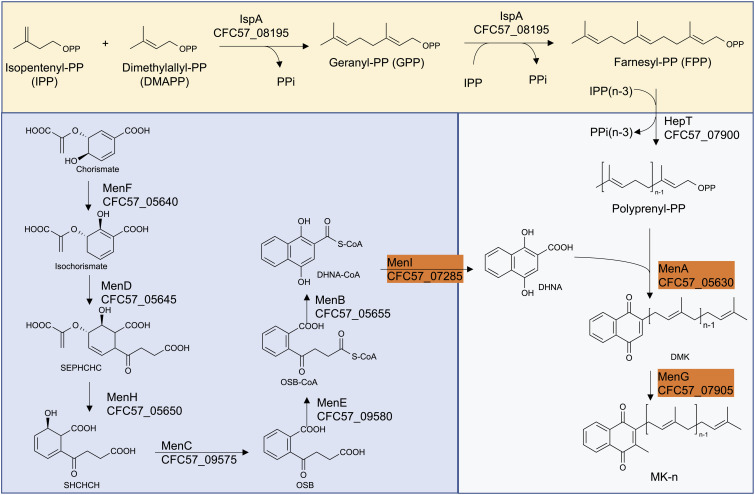
The classical MK biosynthetic pathway in *S. aureus* Smith. The highlighted enzymes have an end to end sequence identity between *S. aureus* Smith, JE2 and Newman strains.

The ability of the Smith strain to produce MK-10 and an association of mutations in MK biosynthetic genes with lysocin E resistance (Hamamoto et al., 2015) triggered us to analyze the MK biosynthetic pathway of this strain so that we could identify the genetic basis of this feature. We obtained the complete genome sequence of the Smith strain using a hybrid Ion PGM and Nanopore MinION sequencing approach (Panthee et al., 2018, 2019). We performed a BLAST search against the genes involved in MK biosynthetic pathway. We found that the Smith strain harbored orthologs of all the genes involved in the classical pathway (Figure 3). We further aligned 11 MK biosynthetic enzymes among Newman, JE2 and Smith strains to find that Newman and JE2 shared an end to end sequence identity in all the enzymes, while Smith strain had amino acid substitution(s) in enzymes except MenA, MenG, and MenI (Figure 3 and Supplementary Figure S2).

Among the Smith MK biosynthetic enzymes that harbored amino acid substitution(s), the majority were involved in the formation of 1,4-dihydroxy-2-naphthoate. Among the enzymes involved in isoprenoid side chain biosynthesis, IspA (CFC57_08195) and HepT (CFC57_07900) had 2, and 3, amino acid substitutions, respectively. IspA is predicted to be involved in the formation of Farnesyl-PP, and HepT is predicted to be involved in the condensation of Isopentenyl-PPs and Farnesyl-PPs, resulting in the formation of all-trans-polyprenyl-PP. Based on this, we speculated that Smith HepT (HepT_Smith_ now onward) might be involved in the formation of longer chain polyprenyl-PPs to be attached to 1,4-dihydroxy-2-naphthoate by MenA (CFC57_05630).

### Analysis of Staphylococcal HepT Involved in Polyprenyl Diphosphate Biosynthesis

**FIGURE 4:**
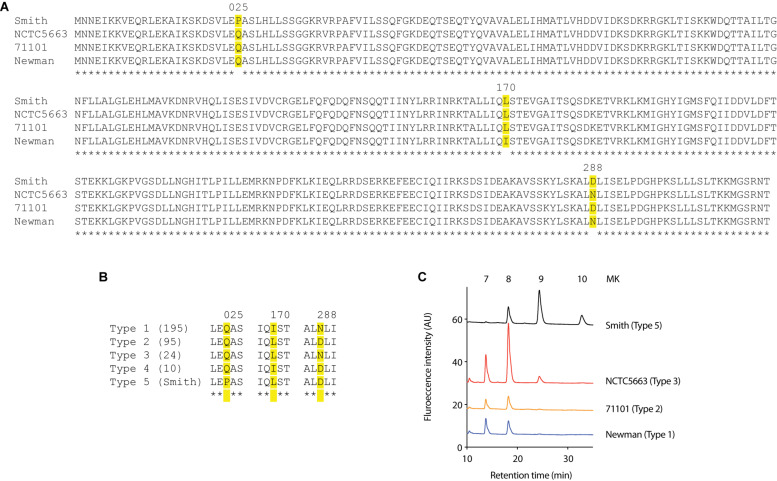
Analysis of Staphylococcal HepTs **(A)** Alignment of HepT from strains Smith, NCTC5663, 71101, and Newman. **(B)** Five types of *S. aureus* based on the position of amino acids at 25, 170, and 288 in the HepT sequence. Numbers in parenthesis indicate the number of strains in each type. Type 5 only contained Smith strain. **(C)** MK content of representative *S. aureus* strains to harbor four HepT types.

We then analyzed the HepT sequence of all *S. aureus* strains whose complete genome sequence was available in NCBI. We focused on three substitutions (Pro-25, Leu-170, and Asp-288) that were different in Smith strain from Newman and JE2 strains (Figure 4A) and found that the HepT from 325 *S. aureus* strains could be categorized to five types which we named type 1 to type 5. Types 1–4 were present in at least 10 strains while type 5 harbored only Smith strain with a Pro-25 (Figure 4B). Among these, we analyzed the MK content from strains harboring four available types of HepT and found that only Smith could produce MK-10 (Figure 4C). This result suggests that Pro-25 of HepT_Smith_ could be responsible for longer chain MK biosynthesis.

### HepT_Smith_ Is Involved in Chain Length Determination of MK

**FIGURE 5:**
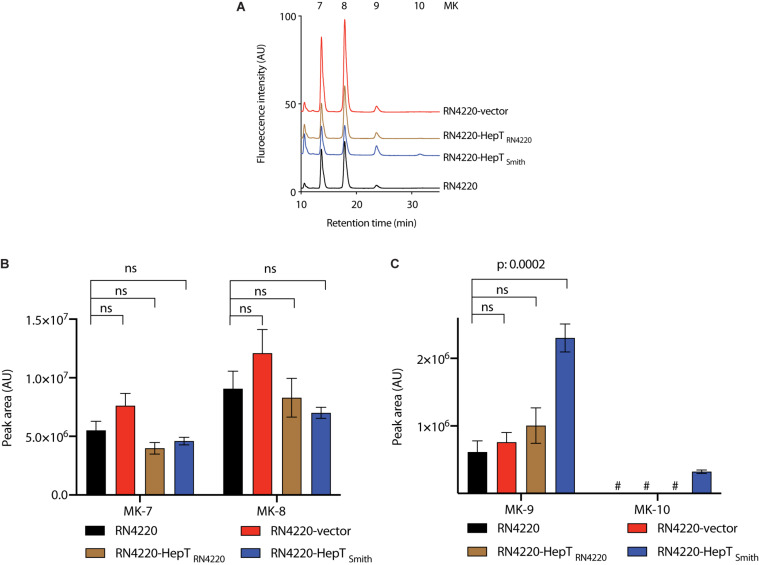
Analysis of MKs from *S. aureus* RN4220 with heterologously expressed HepT. **(A)** Representative HPLC chromatograms. **(B)** Peak area of MK-7 and MK-8. **(C)** Peak area of MK-9 and MK-10. Data are from three independent experiments and represented as mean ± SEM. Statistical analysis was performed by one-way ANOVA using Dunnett’s multiple comparison test in GraphPad Prism, and a *p <* 0.05 was considered significant. ns: non-significant. ^#^Indicates an undetectable amount of MK-10.

To confirm the role of HepT_Smith_ in longer chain MK biosynthesis, we cloned the *hepT* gene from the Smith and RN4220 strains, and expressed under the control of the constitutive expression promoter (Paudel et al., 2020). The plasmids thus obtained were then introduced into *S. aureus* RN4220. *S. aureus* RN4220 is a restriction-deficient mutant derived from strain NCTC8325, and choice of strain for experiments that require genetic manipulation in *S. aureus*. In addition, RN4220 did not produce MK-10, making it an appropriate candidate as a heterologous host to introduce HepT_Smith_ and analyze MK-10 production. We compared the MK production among Smith strain, RN4220 with empty vector, *hepT*_Smith_, and *hepT*_RN4220_. While the production of shorter chain MKs (MK-7 and MK-8) were similar in all the transductants, the introduction of *hepT*_Smith_ in RN4220 resulted in significantly higher production of MK-9 and the appearance of MK-10 (Figures 5A–C). RN4220 harboring empty vector or *hepT*_RN4220_ predominantly produced MK-7 and MK-8, with a trace amount of MK-9, and the MK pattern was indifferent from that of the wild type strain (Figures 5A–C). These results suggest that HepT_Smith_ is responsible for the biosynthesis of longer chain MKs.

### Longer Chain MKs Producing *S. aureus* Are Hypersensitive to Lysocin E

**FIGURE 6:**
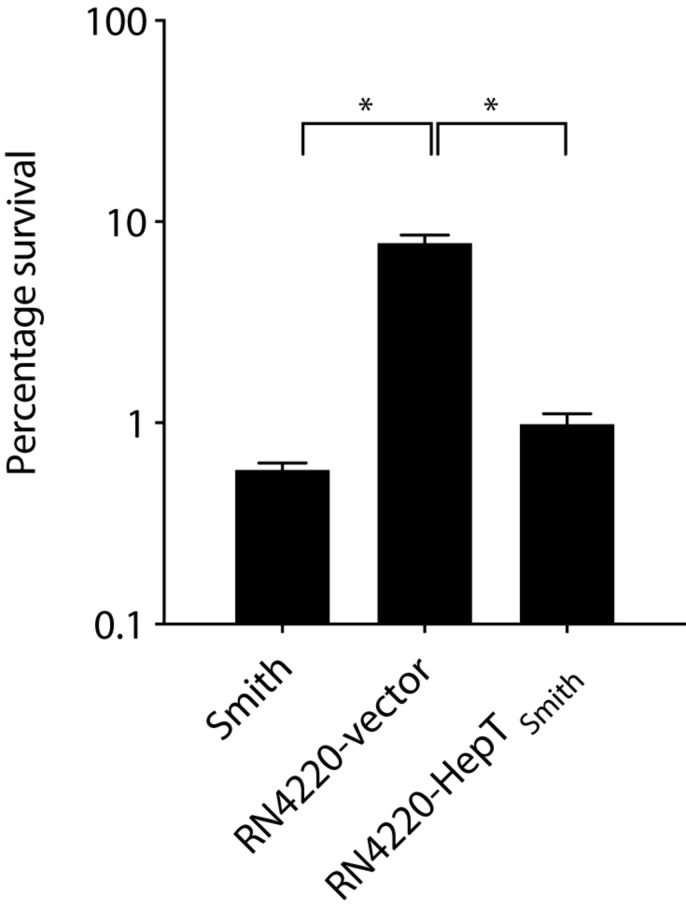
Survival of *S. aureus* in the presence of lysocin E. Exponentially growing bacteria were treated with 1 mg/L of lysocin E for 30 min, and the colony-forming units were counted. Triplicate data are represented as mean ± SEM. Statistical analysis was performed by one-way ANOVA using Dunnett’s multiple comparison test in GraphPad Prism, and the asterisk indicates a *p*-value of < 0.0001.

To elucidate the role of HepT_Smith_ in lysocin E sensitivity, we compared the viability of *S. aureus* Smith and RN4220 strains harboring the empty vector and HepT_Smith_ upon treatment with 1 mg/L of lysocin E. We found that a treatment for 30 min reduced the number of viable bacteria (Figure 6). Furthermore, compared to *S. aureus* RN4220 with empty vector, Smith strain and RN4220 expressing HepT_Smith_ were hypersensitive to lysocin E treatment. Although the sequence of events after the introduction of HepT_Smith_ in RN4220 strain remain to be elucidated, the results obtained indicate that *S. aureus* strains that produce longer chain MKs are hypersensitive toward lysocin E and increased production of MKs harboring longer isoprenoid side chain might be responsible for the phenomena.

In addition to MK biosynthesis, isoprenoids are critical for the biosynthesis of membrane lipids, carotenoids, sterols, and other components of the bacterial cell wall (Oldfield, 2010). Isopentenyl-PP, one of the substrates of HepT and the starting molecule for other isoprenoid biosynthesis, is synthesized either via 2-C-methyl-D-erythritol-4-phosphate (MEP) and/or mevalonate pathway (Lange et al., 2000; Khalid et al., 2017). The enzymes of the MEP pathway have been used as targets for antibiotic discovery against microbes that harbor the MEP pathway (Shigi, 1989; Jomaa et al., 1999). Given that *S. aureus* relies on the mevalonate pathway (Balibar et al., 2009), HepT or other enzymes from this pathway can be targeted for the antistaphylococcal drug development (Desai et al., 2016; Matsumoto et al., 2016).

In *E. coli*, IspB, encoding octaprenyl diphosphate synthase, is responsible for the extension of the isoprenoid side chain (Okada et al., 1997). The crystal structure of *E. coli* IspB has been reported (Han et al., 2015). Two Aspartate rich motifs (DDXXD) are considered crucial for FPP and IPP binding and catalytic activity (Han et al., 2015). The IspB from *E. coli* shows nearly 30% identity to HepT_Smith_ and the P25 lies about 50 AA apart from the first DDXXD. To further elucidate the involvement of P25 in chain-length determination a detailed structural analysis is required.

In summary, we completed the genome sequence of *S. aureus* Smith and performed the genomic analysis of the MK biosynthetic pathway to show that a classical pathway for MK biosynthesis is present in this strain. We demonstrated that Pro-25 substitution in HepT was responsible for longer chain MK biosynthesis, and this was associated with hypersensitivity toward lysocin E. This indicated that lysocin E might disrupt the bacterial membranes containing longer chain MKs more efficiently which requires further analysis. To the best of our knowledge, this is the first report of the identification of *S. aureus* strain producing MK-10.

## Data Availability Statement

The datasets generated for this study can be found in the online repositories. The names of the repository/repositories and accession number(s) can be found below: https://www.ncbi.nlm.nih.gov/genbank/, CP029751, CP029750.

## Author Contributions

SP and HH designed the study. SP and AP performed the experiments and wrote the manuscript. A-CU performed the genome sequence analysis of *S. aureus* 71101. KS integrated the research and critically revised the manuscript for important intellectual content and approved the final version of the manuscript. All authors contributed to the article and approved the submitted version.

## Conflict of Interest

The authors declare that the research was conducted in the absence of any commercial or financial relationships that could be construed as a potential conflict of interest.
